# Can Western water law become more ‘relational’? A survey of comparative laws affecting water across Australasia and the Americas

**DOI:** 10.1080/03036758.2022.2143383

**Published:** 2022-11-27

**Authors:** Elizabeth Macpherson

**Affiliations:** Faculty of Law, University of Canterbury, Christchurch, New Zealand

**Keywords:** Water law, relationality, indigenous rights, constitutional law, comparative law, Australasia, Americas

## Abstract

There is increasing support, in international legal theory and advocacy, for water governance approaches that go beyond the technocratic, and recognise the reciprocal relatedness of water peoples and water places. Such an approach may seem logical within certain Indigenous law and belief systems, but can Western legal frameworks become more ‘relational’? How can they evolve to be capable of meaningfully relating with Indigenous systems of law and governance for water? This article draws on a comprehensive survey of comparative legal developments affecting water across seven settler-colonial countries in Australasia and Latin America that attempt (or profess) to be relational. I critically evaluate these attempts against the ‘yardstick’ of relationality. In each jurisdiction there are unresolved calls for a social, cultural and constitutional transformation of some sort, in which Indigenous and environmental justice are key. The analysis here reveals the potential for constitutional law to drive relational water laws, although without place-based specificity and supporting institutions, resources and redistributions of power, constitutional approaches risk having little practical impact.

## Introduction

This article starts from the position that, when it comes to water, we have a lot of work to do within the legal frameworks developed and administered by Western governments. In Aotearoa, for example, Māori are leading a range of ground-breaking legal and political processes concerning water use and governance.^1^ But there are important, and as yet unresolved, questions about how the Crown’s law, with its colonial history and structures, should *relate to* the rangatiratanga (political authority) of Māori in the governance and use of water (Tau 2017; Ruru 2018b; Te Aho 2019; Taylor et al. 2020; Parsons et al. 2021). These are not questions specific to Aotearoa. Answering them is a common and urgent imperative around the world, as Indigenous peoples in a range of countries continue to be unjustly excluded from Western legal systems that distribute rights to use and manage water, and as the health of waters and related communities continues to decline (Macpherson 2019).

How can Western water law frameworks evolve to be capable of relating with Indigenous systems of law and governance for water?^2^ Specifically, how can they meaningfully respect, reflect and re-enact relationships between water peoples and water places? Resolving these questions will be critical to (at the very least) meet the standard of ‘partnership’ accepted by the Crown as being required under our founding constitutional document, Te Tiriti o Waitangi (The Treaty of Waitangi)*,* and the legal directives of a range of international instruments the New Zealand government has already signed up to*.* But there is also now an overwhelming body of international scholarship and advocacy calling for urgent changes to environmental law frameworks if we are to overcome extinction-level threats to global environmental futures (see, e.g. May and Daly 2015; Kotzé 2020). As will be discussed, much of this advocates for a shift to governance approaches predicated on interdependent, co-constitutive relationships between humans and nature, which fairly ‘engage’ Indigenous peoples and their rights, interests and values (see, e.g. Intergovernmental Panel on Climate Change 2022, p. 64; Intergovernmental Science-Policy Platform on Biodiversity and Ecosystem Services 2019, pp. 14–16; Intergovernmental Science-Policy Platform on Biodiversity and Ecosystems Services 2022, p. 34).^3^

In this article I ask: can Western legal frameworks bearing on the use and governance of water become more ‘relational’? The ‘relational turn’ has now captured the attention of legal scholars, having a much longer tradition in the social sciences where multiple different theories of relationality have been, and continue to be, developed (Haraway 2016; de la Cadena and Blaser 2018; Escobar 2018; Grear et al. 2021; Jackson et al. 2022). As I will discuss, the younger, socio-legal scholarship framed around relationality has substantive and procedural implications – being broadly based on ideas of (often kinship-based) relatedness, relationships and ‘belonging’ between people and place (Clark and Page 2022, p. 65; Watson 2022). In terms of substance, it tends to understand humans as related to and within holistic, living, ecosystems, in the context of complex systems of reciprocal and intertwined rights and responsibilities of use and care (Macpherson 2022). Such an approach can be contrasted with typical Western laws, where humans are perceived as separate to nature, and law’s engagement with nature is fragmented across seemingly arbitrary jurisdictional scales (Clark and Page 2022, p. 29; Macpherson 2022). Often, but not always, Indigenous ontologies are referred to in support of relational framings of environmental law (Arstein-Kerslake et al. 2021; Fisher et al. 2022; Watson 2022). In terms of process, relational understandings of law depart from static notions of law and rights to focus on the processes of relationships, dialogue and negotiation in plural, multicultural legal settings (Davies 2017). As such, relational legal theories usually emphasise the importance of place-based decision-making and governance for related ecosystems, again often highlighting the need for states to embrace Indigenous worldview and law (RiverOfLife et al. 2020, 2021b).

In this article I adopt an understanding of relationality, when applied to the content and scope of water law, that invokes the relatedness between water peoples and water places, performed through complex systems of rights and responsibilities. But relationality also entails the co-constituting processes of multiple peoples and systems of law relating to each other (Yazdiha 2017, p. 5), including, relevantly, the law of (settler) governments, Indigenous peoples, and water itself. As Blomley (2016, p. 251) has argued, ‘a relational view rejects the idea of space as outside of the processes that define it, including the actors who imbue it with meaning’. Relationality, thereby, goes to the heart of power relations between peoples (Tănăsescu 2022, p. 17), and has been accompanied by calls to accommodate legal pluralism and place-based power-sharing, including through partnership between settler governments and Indigenous peoples in designing and implementing the legal frameworks that determine water access, use and governance (Coates 2018; Ruru 2018a, 2018b; Taylor and Hikuroa 2022).

In considering how Western legal frameworks affecting the use and governance of water can become more relational, I look beyond Aotearoa’s shores, to other nation states and Indigenous nations grappling with similar questions. For this article, I conducted a comprehensive survey and critical analysis of recent legal developments happening in seven countries in Australasia and the Americas: Aotearoa, Australia, Colombia, Mexico, the United States, Canada and Chile. All settler colonial states with Indigenous populations, these countries have been sites of legal and political contestation and renewed energy around water reform and justice, including at the constitutional level.

As a comparative legal scholar, I believe that there are always lessons to be learned (both positive and negative) from casting an eye broadly towards the experiences of peoples facing similar challenges in other places; provided that close attention is paid to the determining role of local cultural, political and historical context (Cotterrell 1995; Orucu 2006). Comparative experience is often hard to access, because of cultural and linguistic barriers, but this article draws on 20 years of practical legal and research experience on Indigenous water rights in comparative law, including in-depth socio-legal investigations and fieldwork in these countries during the past decade.^4^

I use the survey in this article to highlight attempts at designing and implementing relational water laws emerging from recent legal reforms and judicial decisions across the seven jurisdictions, revealing important lessons for the challenge of a more relational water law in Aotearoa. There is still ‘much to be done’ (Coates 2018, p. 181), but in comparative experience in the last few years, there have been a few legal advances that are worth emphasising.

The article is structured as follows. In part 2, I consider the theoretical case for taking a relational approach to water law in the international and domestic socio-legal and interdisciplinary scholarship. Part 3 sets out the results of the survey of comparative water laws, highlighting certain legal developments that reflect or support ideas of relationship and relatedness between (multiple) water peoples and water places. And in the fourth and final part of the article, I draw findings from the comparative analysis. I use the comparative study to identify seven legal characteristics associated to varying degrees with relational water laws across the seven jurisdictions, and attempt to evaluate the degree to which each is implicated in Table 1. These are the extent to which the law reflects the relatedness of people and place; whether the law depends on constitutional rights protections; the extent to which the law reflects or defers to Indigenous law; the degree to which the law provides for representation arrangements; the effectiveness of institutions and collaborations to implement the law; and the extent to which the law enables power-sharing with Indigenous peoples. The analysis reveals that the most relational examples of water laws are those where legal and institutional arrangements supporting them not only reflect the relatedness of water peoples and places in Indigenous ontologies and epistemologies, but allow for the sharing and transfer of state-assumed power.

**Table 1.** A simplistic evaluation of Western laws’ efforts to relate to rivers and river peoples.

	Law	Relatedness	Place-based	Constitutional	Indigenous law	Representation	Institutions	Power-sharing
Aotearoa	Te Awa Tupua	High	Yes	Low	High	High	High	Medium
	Te Mana o Te Wai	High	No	Low	Medium	Medium	Medium	Low
Australia	Birrarung (Yarra River)	High	Yes	Low	Medium	Low	Low	Low
	Water is Life	High	No	Low	Medium	Medium	Low	Low
Colombia	Atrato River	High	Yes	High	Medium	Medium	Low	Low
Mexico	Rights of nature	Low	No	High	Low	Low	Low	Low
United States	Rights of nature	Low	Yes	Medium	Low	Low	Low	Low
	Tribal rights of rivers	High	Yes	Medium	High	High	Low	Low
Canada	Muteshekau-shipu (Magpie)	High	Yes	Medium	Medium	Unclear	Unclear	Unclear
Chile	‘Green’ Constitution	High	No	High	High	High	High	High

## Relating to water: rights, responsibilities and reciprocity

The Matike Mai Report on constitutional transformation in Aotearoa, led by Mutu and Jackson, posited the idea of three spheres of influence as a potential model for understanding the constitutional relationship between Tangata Whenua (Māori, people of the land) and Tangata Tiriti (settlers) (The Independent Working Group on Constitutional Transformation 2016).^5^ This model has been affirmed, more recently, in the He Pua Pua Report on the plan to realise the United Nations Declaration on the Rights of Indigenous Peoples (Charters et al. 2019). It has also been applied to the specific context of water by Taylor and Hikuroa (2022), in considering how the Crown can relate to Māori in the governance and use of water. The three spheres of influence model shows how Māori iwi and hapū (tribes and subtribes) with guaranteed rights of rangatiratanga or political authority over their territories and people guaranteed under article 2 of Te Tiriti (Mikaere 2007, p. 54, citing Moana Jackson), engage in a joint or relational sphere of influence with the Crown. The Crown, in turn, operates within the Kāwanatanga (Crown governance) sphere. The middle sphere is expressly called ‘the relational sphere’, based on a relationship of partnership between the Crown and Māori, hopefully with all parts working together towards a common vision.

Moving towards a more relational water law in Aotearoa would be a significant challenge for multiple reasons. These include that Crown law (indeed Western, colonial law everywhere in the world) has not historically framed its interaction with Indigenous peoples as one of partnership, rather one of concerted effort of dominance of its sphere over the other (Macpherson 2019). Accordingly, it is critical to consider how Western water law can ‘come to the party’. Cribb et al. (2022), in a recent paper about implementation of the ground-breaking Te Awa Tupua model for the Whanganui river, refer to this as ‘the new (but old) consciousness needed to implement Indigenous frameworks in non-Indigenous organisations’. They argue, in that context, ‘that a paradigm shift is necessary for non-Indigenous organisations to view human-nature relationships through an Indigenous lens’. Parsons et al. (2021, p. 477) in their work on decolonising blue spaces similarly refer to needing ‘such new (or some would say old) ways of thinking about and enacting respectful inter-being relationality’.

Relationality also helps us to understand how people (including, but not limited to, Indigenous peoples) relate to water places, to form a basis for water authority, use, rights and governance. There is increasing support for relationality as a conceptual framework for legal and governance frameworks applying to nature, ecosystems or the environment, especially in conjunction with Indigenous ontologies and epistemologies (de la Cadena and Blaser 2018; Escobar 2018; Ma Rhea 2018; Dudgeon and Bray 2019; Fisher and Parsons 2020; Milgin et al. 2020; Ulloa 2020; Vásquez-Fernández and Ahenakew pii tai poo taa 2020; Poelina et al. 2021; Reddekop and Trownsell 2021; Reid 2021; Tynan 2021; Lindstrom 2022).

As explained above, the focus of a relational approach to water law and governance is the relatedness and belonging of water peoples to water places (Arstein-Kerslake et al. 2021; Clark and Page 2022; Macpherson 2022; Tănăsescu 2022). Muru-Lanning (2016, p. 142) explains, through her research on the Waikato river, how the river peoples’ Tikanga (law) centres around their relationship with their Tupuna Awa, or ancestral river, and the fact that they belong to the river. In this place-based relational approach, the mana (or authority) of people is of central importance, and ‘responsibilities’ (Martin 2018) to care for rivers go hand in hand with rights like ownership (Te Aho 2019).

Relational approaches to water are typically framed around ‘place-based’ (Bartel 2018) relatedness or kinship connections between peoples and ‘living waters’ (Ruru 2013, 2018b; Lyons and Barber 2021; RiverOfLife, et al. 2021a; Wooltorton et al. 2021; Laborde and Jackson 2022); in Aotearoa, through whakapapa (Williams 2013). In the recent Supreme Court *Trans-Tasman Resources v Taranaki-Whanganui Conservation Board* decision, which (amongst other things) considered the effect of Tikanga in the context of seabed mining consents, Williams J used the word ‘relational’ when explaining iwi interests in the case (2021[109]):

I would merely add that this question must not only be viewed through a Pākehā lens … . As the Court of Appeal rightly pointed out, the interests of iwi with mana moana in the consent area are the longest-standing human-related interests in that place. As with all interests, they reflect the relevant values of the interest-holder. Those values—mana, whanaungatanga and kaitiakitanga—are relational.

Because of the place-based relatedness of peoples and waters, and the reciprocity between them, people rely on water to provide benefits like food, water, transport, irrigation and economy, as well as having an obligation to care for water (Te Aho 2018; Taylor et al. 2020; Bédard 2021; Moggridge and Thompson 2021). As such, humans are positioned within and interdependent on living ecosystems in a reciprocal exchange (Fisher and Parsons 2020). This is quite a different approach to that taken in conventional Western laws (Dehm 2022), which have routinely ignored human-water relationships. For example, in Western water law, water is routinely perceived as merely a resource to be shared amongst commercial interests for extractive utility, without reciprocal obligations of care (Macpherson 2019). Similarly problematic, in Western conservation law humans are typically perceived as existing independent of nature (Demeritt 2002), while nature is seen as scenery to be cordoned off from human extractive use, in things like protected areas (see Lyver et al. 2019). Sometimes called ‘environmental colonialism’ (O’Donnell et al. 2020), this Western conservationist approach to nature has had catastrophic impacts on Indigenous peoples throughout the world who have at times, as a result, been enclosed out of traditional territories and livelihoods (McCormack 2021). Stensrud explains, in the context of Andean Peru, that ‘[w]ater is not an abstract resource that can be separated from the relational world in which it partakes’ … ‘Political struggles to defend water resources in the Andes are not motivated by desires to recover a “pure nature” separate from human activities. The farmers in Colca Valley rather want to have a role in the making of the environment where they make their livelihoods’ (Stensrud 2016, pp. 93–94). Nor does nature need to be pristine or conserved to be loved; in fact, if a relative is sick, one should take more care to look after it.

Relational thinking helps us embrace legal pluralism (Gutmann 2021), or the existence of multiple intersecting systems of law in one place at one time (Jackson 2021), such as Crown law and Tikanga (Williams 2021), and to focus on establishing processes and institutions that support relationships in between (Durie 2018; Macpherson 2022). This means that relational approaches to water law and governance cannot only be static declarations of rights or entitlements. Their focus is also necessarily on the ongoing practice and co-constitutive performance of relationships between beings, through process, protocol and ritual (Manikuakanishtiku et al. 2022; Tănăsescu 2022). Gover explains that the purpose of relational laws is, ‘not to arrive at a state of finality in the design of formal arrangements, but to create the conditions for an open, inclusive and on-going dialogue’ (Gover 2009, p. 8). Clark and Page (2022) describe a relational legal conception of ‘the lawful forest’ as ‘dynamic, performative, relational, co-constituted and inherently social’.

Relational approaches to water laws are, therefore, more dynamic and responsive than typical Western laws, which are fixed in time and space; subject to definitional boundaries that cut through water relationships and beings – imposing arbitrary sectorial or jurisdictional divisions (Macpherson, Urlich, et al. 2021). Designing water laws and governance arrangements for ‘the pluriverse’ (Escobar 2018; Viaene 2021) would seem to require this level of flexibility and adaptability, where ‘[d]uties and responsibilities can and do move between times, spaces, realms, and forms and allow for flexibility in usage but always with the emphasis being on maintaining balance and life across generations and beings (maintaining the mauri (life force) of the river, the people, plants, animals, and others)’ (Parsons et al. 2021, p. 477).

Relational approaches to water law are also better aligned to the demands of adaptive (Cosens and Chaffin 2016), ecosystem-based (Hewitt 2018), collaborative (Holley and Sinclair 2018), participatory or bottom-up (Easterly 2008) and nature-based governance (Krauze and Wagner 2019), which are all increasingly influential in international environmental theory. This is apparent from Talbot-Jones and Bennett’s recent analysis of the political-institutional framework for Te Awa Tupua, recognised in 2017 as a legal person as part of a relational governance model devised for settlement of Whanganui river claims under Te Tiriti. They explain how the approach: ‘transformed the institutional governance arrangement from one that was prescriptive, fragmented, adversarial, and characterised by a failure to reflect the values shared by the community – symptoms of a poorly designed top-down system – to one that is sensitive to local knowledge and contexts and provide for the direct participation of local actors – favourable characteristics of a bottom-up system’ (Talbot-Jones and Bennett 2022, p. 66).

If we accept, as I maintain, that relational water law is about both substance and process, it becomes very important to define the legal entities or persons, institutions and arrangements that will support the practice of water relationships in a way that balances power asymmetries (Tănăsescu 2016, Fisher et al. 2022). Talbot-Jones and Bennett explain how Te Awa Tupua legislation, ‘introduced new actors to the operational level of decision-making and granted them decision-making authority. A further collection of actors and groups was created to scaffold the new legal rights arrangement and help consolidate a shift in decision-making responsibility from the policy level to the operational level of the institutional arrangement’ (Talbot-Jones and Bennett 2022, p. 74). Consequently, a relational approach to water law will necessitate the sharing or shifting of power between the legal entities or persons necessary to enact relational water law (Arstein-Kerslake et al. 2021). In a settler-colonial context, these persons are the Indigenous authorities with place-based authority or manawhenua (rangatiratanga sphere), the Crown or state (kāwanatanga sphere), and potentially even water persons or beings themselves. As the countries highlighted in this article show, without effective, fair and properly-resourced institutions and power-sharing arrangements, there can be no truly-relational water laws (O’Donnell and Macpherson 2018; Coombes 2020).

## Relational water laws – highlights from Australasia and the Americas

### Aotearoa

Probably the leading global example of a relational approach to water law is Aotearoa’s Te Awa Tupua model.^6^ Te Awa Tupua was recognised by legislation in 2017 to be a legal person as part of a Tiriti settlement between the Crown and the Whanganui River iwi (Te Awa Tupua (Whanganui River Claims Settlement) Act 2017). The iwi relate to the river as a living ancestor (Waitangi Tribunal 1999, p. xiii; Salmond 2017, p. 300; Cribb et al. 2022), and have maintained their claims to authority, ownership and control of the river since prior to colonisation (Waitangi Tribunal 1999, p. 55–56, *In re the Bed of the Wanganui River* 1962). The Waitangi Tribunal inquiry into the iwi’s river claims documented a range of place-based impacts including the obstruction of mahinga kai (food gathering areas and practices), navigation and ceremonial uses by Crown-authorised activity in the catchment, such as agriculture, urbanisation, resource extraction and hydroelectric development. Iwi ownership, control and authority over river governance were undermined by the vesting of river ownership and regulatory power (unilaterally and without compensation) in the Crown (Waitangi Tribunal 1999, p. 55–56),^7^ which is now exercised by local authorities under the Resource Management Act 1991 (RMA), under the broad purpose of ‘sustainable management’ (Resource Management Act 1991 s. 5).

Whanganui iwi resistance to the assumption of ownership and control of the river by the Crown and local government is longstanding, including multiple court cases, an in-depth Waitangi Tribunal River Inquiry (Waitangi Tribunal 1999, p. 337),^8^ and eventual negotiation and settlement culminating in the Te Awa Tupua (Whanganui River Claims Settlement) Act 2017. This legislation recognises the status of the river (and its tributaries) as ‘an indivisible and living whole, comprising the Whanganui River from the mountains to the sea, incorporating all its physical and meta-physical elements’ (s 12). It sets up a framework for river governance called Te Pā Auroa (Cribb et al. 2022), which comprises a range of legal entities involving iwi, hapū, government and community and recreational interests (ss 27–35), working under a common set of fundamental values called Tupua Te Kawa (s 15). These acknowledge the direct link between the health of the river and the health of the people, emphasise the living, integrated and holistic nature of the river, and acknowledge the responsibility of the Whanganui iwi to work collaboratively with other river interests to further the river’s health and wellbeing (s 13(a)). The model also recognises the living being status of the river as a ‘legal person’^9^ with all corresponding rights, powers, duties, and liabilities (s 14), as represented by Te Pou Tupua (the human face of the river) (ss 18–19).^10^

Te Awa Tupua Act has put Whanganui on the map, with intense interest from international media and scholarship (Kauffman and Martin 2021), much of which seeks to position the legislation as aligned to the global ‘rights of nature’ movement. This is curious, given that New Zealand law does not directly recognise any environmental rights, let alone the rights of nature, within the Constitutional framework (Macpherson, Borchgrevink, et al. 2021). More accurately, in Whanganui, the legislation recognises the relationship between river iwi and Te Awa Tupua (Cribb et al. 2022; Te Aho 2014), the legal person model being one creative strategy within the broader Pā Auroa model, to influence government decision-makers to prioritise river and river people, via a legal approximation from Western law that is familiar to the Crown. The Awa Tupua model is an attempt to resist the carving up of the river system by Western legal frameworks – into discrete legal and property regimes for bed, banks, adjacent land, minerals, wildlife and resources. Positioning the river as a living being and ancestor would seem inconsistent with such a fragmented approach. Who could cut a relative up this way? The model therefore allows for holism and integration in river governance, something sought by (but allusive to) Western legal frameworks, whereby river managers consider the impacts of decision making throughout the river system, including on the people connected to it. The model centres Tikanga Māori in reflecting relationships between people and the river but brings other river users in conversation with the model via their participation in collaborative governance arrangements.

As Coates points out (2018, p. 169), the Awa Tupua model represents a ‘subtle and incremental shift in the Crown–Māori constitutional relationship’ (See also Sanders (2018), p. 231; Tănăsescu (2020), p 446). But it is still a political compromise arising out of a Tiriti settlement (Te Aho 2019, p. 1618), and (as such) is limited by the extent to which the Crown agreed to its parameters (Jones 2016, p. 98). For example, the legislation does not go so far as to transfer full political authority or ownership over the river to the iwi.^11^ Its influence (and that of other river settlements) can be seen in the National Policy Statement for Freshwater Management’s ‘Fundamental concept’, Te Mana o Te Wai*,* which guides regional authorities in the management of water under the RMA (New Zealand Government 2020). Te Mana o Te Wai sets out values that seek to recognise the holistic and living nature of water (13[3.5]), and the relatedness of people with water (5[1.3(4)]), and prioritises the health of the water ahead of other economic, social or cultural interests (6[1.3(5)], 9[2.1]). Yet, some have criticised Te Mana o Te Wai for being a Crown concept used to appease, appropriate and essentialise Māori, thereby drawing attention away from more radical political-structural claims (Taylor 2022). Kitson and Cain (2022) emphasise that Te Mana o te Wai needs to be redefined on local terms, to align to a place-based Māori worldview. These conversations highlight the risk and difficulty of integrating concepts from Te Ao Māori into a non-Māori system (Taylor 2022). Moreover, as the Crown charges ahead with resource management reform, concerns remain about the ongoing failure to settle substantive Māori water claims and the impact of national water reforms on hard-fought, place-based settlements (Natural and Built Environments Bill 2021 (Exposure Draft)).

### Australia

Australian laws have historically taken a technocratic approach to water resources management, with a focus on the market or engineering uses of water (Crase 2011; Horne and O’Donnell 2014; Fisher 2016; Garrick et al. 2018) in almost complete ignorance of the relationships people have with rivers and other water bodies (Jackson 2021). This might be explained, in part, by a reluctance amongst Australian governments and courts to recognise the rights Aboriginal and Torres Strait Islanders hold to water (Hartwig et al. 2021, 2022; Wardle 2021; O’Donnell et al. 2022) in the context of weak environmental constitutionalism. For example, it is very difficult for Indigenous groups to make out ‘native title’ claims for substantive water rights for any sort of consumptive or extractive purposes under the Native Title Act (Native Title Act 1993 ss. 223-225; Mabo 1992, p. 59 (Brennan J)), a limiting approach that has reverberated through state-based water laws and planning policy (Macpherson 2019; O’Donnell et al. 2022). Consequently, Indigenous peoples have been essentially ‘locked out’ of water allocation frameworks, in the context of fierce competition for water by other users. However, with increasing preparedness to hear Indigenous demands (Referendum Council 2017), and general growing acceptance of the socio-cultural elements of water use reflected in ideas like ‘cultural flows’ (Provis 2021), there are now some examples of Australian laws that are more relational.

The leading example of a relational water law in Australia is the Yarra River Protection (Wilip-gin Birrarung murron) Act 2017 in Victoria, which recognises the ‘intrinsic connection’ of Traditional Owners to the Yarra River and its Country, as custodians. The Act recognises the Yarra as a ‘living and integrated natural entity’ (s 1) and appoints the Birrarung Council to advise the Minister and to advocate for protection and preservation of the river (s 46). The Act also sets out ‘protection principles’ for decision-makers to consider in river planning (pt 2) and provides for the development of strategic river plans (pt 4), addressing a range of environmental, cultural and recreational interests. Despite the Victorian Government having committed to Aboriginal self-determination and a truth-telling and Treaty process, the Birrarung Act is not a partnership model, being State-led and only providing for two Aboriginal representatives on the Council (O’Bryan 2017). Similar legislation applies to a peculiar ‘ecosystem’; the Great Ocean Road and Environs Protection Act 2020 (Vic), and, at the policy level, ‘action plans’ for the waterways of the west and rivers of the Barwon also recognise rivers as ‘living, integrated entities’ (State of Victoria 2021a, 2021b). It appears, at least in Victorian policy-making, that river relationality is on trend.

In other parts of Australia, Indigenous peoples are fighting for recognition of their substantive rights to access water and their inherent rights to govern waters according to their own laws (North Australian Indigenous Land and Sea Management Alliance 2009; Jackson et al. 2021). An example comes from the Maruwarra (Fitzroy) river (Hill et al. 2022; Wooltorton et al. 2021), recognised in the Fitzroy River Declaration as ‘a living ancestral being’ with ‘a right to life’ (RiverOfLife et al. 2021b, p. 2021), although not yet formally recognised by the state. The Declaration provides that the river ‘must be protected for current and future generations, and managed jointly by the Traditional Owners of the river’ (Fitzroy River Council 2016). In other places, there is increasing emphasis on ‘partnership’ with Aboriginal peoples in water management practices (Jackson and Nias 2019).

In Victoria, the State has taken steps towards improving recognition of Aboriginal water values and representation in water governance (State of Victoria 2016, p. 140; Water and Catchment Legislation Amendment Act 2019 (Vic)), and redistributing ‘economic water rights’ to Aboriginal people. Victoria’s 2022 roadmap for Traditional Owner access to water is called ‘Water is Life’ (State of Victoria 2022). The Roadmap proposes a ‘restorative justice approach’, which presents options to return water to Traditional Owners,^12^ and recognise their role as water managers. Water is Life claims to be framed around a partnership approach, including dual government policy commitments and a collection of place-based Nation statements written by Traditional Owners across Victoria, detailing their objectives for water (State of Victoria 2022). It seeks, as a targeted outcome of the Roadmap, ‘[r]ecognition of waterways and their surrounding land as living, integrated, natural and cultural entities in law, with Traditional Owners recognised as a unique ‘voice’ of the living entities’ (State of Victoria 2022, p. 26). It is too early to assess the impact of the Roadmap, which includes a disclaimer that the Nation statements ‘are not Victorian Government commitments’ (State of Victoria 2022, p. 20). At best, Victoria may signal a new Australian standard around the recognition of plural law-making and governance functions for the State and Aboriginal nations. However, a sceptic might wonder if the Victorian developments are merely another attempt to incorporate Indigenous rights and interests in State-designed and mediated water law systems (Hartwig et al. 2021, 2022).

### Colombia

Colombia appears to be the most prolific producer of seemingly relational water laws, with dozens of rivers now recognised as living beings and ‘subjects of rights’, sometimes under the care or guardianship of Indigenous, local or Afrodescendant communities. The cases have invoked Colombia’s ‘Green Constitution’ (Navas 2002), which protects environmental, Indigenous and Afrodescendant rights,^13^ and contemplates special relationships with territory based on ethnic identity, difference and culture (Lemaitre 2009).

The first and most well-known Colombian case concerns the Atrato River, recognised by the Constitutional Court in 2016 as a ‘subject of rights’ (*Centro de Estudios para la Justicia Social ‘Tierra Digna’ y otros v Presidente de la Republica y otros* 2016).^14^ The case was presented on behalf of Afro-descendant and Indigenous communities that live alongside the Atrato, and sought a declaration that their constitutional rights to life, health, water, and healthy environment had been violated as a result of damage to the river from illegal mining (González Serrano 2020; Macpherson et al. 2020). The Court recognised the ‘biocultural rights’ of the communities, based on an inseparable relationship between river people and river, perceived as a living entity and giver of life (Bavikatte and Bennett 2015). The Court describes the river communities’ right as one ‘to administer and exercise trusteeship in an autonomous manner over their territories – in accordance with their own laws and customs’ (*Tierra Digna* 2016, p. 43).

The Court found that the Colombian State had breached all the constitutional human rights protections alleged by failing to protect the river and its communities. Further, the Court recognised the Atrato River, its basin and tributaries, as an entity subject of rights of protection, conservation, maintenance, and restoration by the State and ethnic communities (*Tierra Digna* 2016, p. 43). The Court made several orders for the realisation of those rights: setting up a commission of guardians for the river; a panel of experts to assist the guardians; an integrated watershed management governance body comprised of national and regional administrative authorities; and a commission for the eradication of illegal mining and deforestation. In doing all this, the Court went beyond anything raised in counsel’s submissions and referred to rights of nature protections in Ecuador and Bolivia.^15^ The Court even cited Te Awa Tupua Act (and the legislation recognising Te Urewera as a legal entity with rights), explained by the simple curiosity that the Judge’s clerk in the case had visited Aotearoa during postgraduate research, becoming fascinated by the Whanganui River settlement (Macpherson 2019).

The Atrato case, and those that have followed it (Macpherson et al. 2020), continue to divide opponents and supporters, with research noting poor implementation of the case in Colombia’s complex social-ecological and security context (Macpherson, Borchgrevink, et al. 2021; Richardson and McNeish 2021). In some respects, it carves out a space for the Indigenous and Afrodescendant communities to once more exercise autonomy over a river as guardians. However, although the Colombian river cases generally acknowledge Indigenous water relationships,^16^ they do nothing to disrupt colonial distributions of water under the Colombian Civil Code, where water is a ‘bien de uso publico’ (public good), belonging to all the nation, while the State has the right of ‘dominio’, to use enjoy and dispose of water as it sees fit (see Colombian Civil Code 1887 art. 674). In a country where 70 per cent of small water users rely on ‘informal’ or customary use rights with no formal water concession, large elites have accumulated formal rights, reinforcing the exclusion of local and Indigenous users and encroaching on their territories (Roa-García et al. 2015, p. 274). Nowhere in the Colombian water laws is there any specific provision for Indigenous peoples to use, own or govern water on their territories (Defensoría del Pueblo de Colombia 2012, p. 16).

### Mexico

Mexico has a similar tradition to Colombia in the recognition of rivers (or nature broadly) as subjects of rights, although Mexican developments are less-known to an international audience. Mexican constitutional law also recognises environmental rights, both at State and Federal level,^17^ including the rights to water and a clean and healthy environment (Constitución Política de los Estados Unidos Mexicanos, art. 4). However, Mexico is plagued by serious water quality and access issues (United Nations Human Rights Council 2017, p. 6) and enduring disputes around Indigenous water rights (Estrada-Guevara 2017, p. 72; see also Casanova et al. 2001; Rivas 2014, p. 133; Radonic 2017, p. 151, 152). This context has provided fertile ground for the recognition of rights of nature (and rivers) in Mexico.

The first legal development happened in 2014, when the Guerrero State Constitution was amended to ‘guarantee and protect the rights of nature’ based on ‘a respect for life in all its manifestations’ (Constitución Política del estado de Guerrero 1917 art. 2). A similar provision was included in the Constitution of the newly formed State of Mexico City in 2017, providing that the right to a healthy environment should be elaborated in a secondary law ‘with the objective of recognising and regulating the broad protection of the rights of nature and all its ecosystems and species as a collective entity legal subject’ (Constitución Ciudad de México 2018 art. 13 A 3). Also in the State of Mexico City, the Environmental Law for the Protection of the Earth provides that ‘[t]he Earth is a living and dynamic system formed by the indivisible community of all life systems and living beings, interrelated, interdependent and complementary, that share a common destiny’ (Ley Ambiental de Protección a la Tierra del Distrito Federal 2000 art. 86 bis 1). In 2017, the Constitution of the State of Colima was amended to recognise that ‘[n]ature, comprised of all its ecosystems and species as a collective legal subject, must be respected in its existence, in its restoration and in the regeneration of its natural cycles, together with the conservation of its ecological structure and functions, as established by law’ (Constitución Política del Estado Libre y Soberano de Colima 2017 art. 2, IX(a)). In 2021, the State of Oaxaca also inserted a rights of nature provision in its Constitution, including ‘the right to preservation, the right to protection of its elements, the right to exercise its vital and natural cycles and its ecological functions, the right to integral restoration of its ecological balance, and the right to be legally represented’ (Constitucion Politica del Estado Libre y Soberano De Oaxaca 1922).

Mexico has also been the site of activism towards recognising the rights of specific rivers ‘to survive and thrive’.^18^ In particular, community activism for protection of the highly contaminated Magdalena river, known as the ‘last living river in Mexico City’ (Montoliu and Magrinyà, p. 37), spearheaded the inclusion of rights of nature in the Mexico City State Constitution. The US-based Earth Law Centre continues to work with local organisations and citizens to advocate for the rights of the Magdalena and other rivers through a ‘Declaration of River Rights’ in Mexico (Earth Law Centre [date unknown]).

However, despite significant Indigenous population in Mexico, with constitutionally protected territorial rights, the voices of Indigenous peoples are conspicuously silent in the Mexican rights of nature debate (Macpherson 2021). The broad declarations of rights of nature have also been poorly elaborated, without supporting laws or regulations required for their implementation.^19^ To an extent, these protections recognise human relationality with water, but they have largely been driven by American environmental NGOs and Mexican environmental politics (Garza Grimaldo 2015), and reflect national and international advocacy around environmental rights and the rights of nature rather than place-based or Indigenous realities. As such, they tend to emphasise Western-style rights of a broad and amorphous ‘nature’ to survive in isolation from (or in spite of) humans, rather than reciprocal interrelatedness or kinship between particular peoples and specific rivers.

### United States

The United States is home to some of the earliest legal attempts to recognise the rights of rivers. Many of these have been driven (again) by large environmental NGOs, but some have been led by First Nations groups. In the US, Indigenous domestic-dependent, sovereign nations (Fletcher 2018; International Working Group for Indigenous Affairs 2019) may have reserved water rights arising from historical treaties (*Winters v United States* 1908; The McCarran Amendment; Brougher 2011; Fletcher 2018), and exclusive land and resource rights under the common law doctrine of ‘Indian title’ (*Johnson v McIntosh* 1823). However, the vast majority of Indigenous reserved lands are in the western, arid States, where access to water is highly competitive. The practical situation in terms of Indigenous water access in any place depends on a complicated mix of constitutional law, treaties, agreements, legislation and court decisions (Curley 2019, 2021; Ellis and Perry 2020; Macpherson 2021).

Although US developments do not rest on constitutional environmental protections, they also have a constitutional flavour. Rights of nature advocacy has responded to a lack of a general public interest ‘standing’ (Stone 1972) under Article III of the US Constitution, requiring that claimants taking matters to the court on environmental grounds must prove that they have directly suffered an injury (Babcock 2016, p. 6).^20^ As a result, a cascade of municipal ordinances protecting the rights of nature or ecosystems to ‘exist and thrive’ have been made since the year 2000, some involving pollution in rivers or water contamination through fracking.^21^ Attempts at City and State jurisdictional level to recognise the rights of the Colorado River and Lake Erie have been met with fierce, constitutional opposition (*The Colorado River Ecosystem and Deep Green Resistance et al. v State of Colorado* (Order to Dismiss); *Drewes Farms Partnership v City of Toledo*, *Ohio* (Preliminary Injunction Order); Hedden-Nicely and Caldwell 2020).

Like rights of nature developments in Mexico, the US cases have been primarily driven by environmental activists, and do not centre human relationality with rivers. However, some First Nations groups have used their jurisdiction as domestic-dependent, sovereign nations to pass their own rights of nature laws.^22^ In 2015; the Ho-Chunk Nation General Council incorporated the rights of nature in its Constitution (The Constitution of the Ho-Chunk Nation [date unknown]), and in 2017, the Ponca Nation in Oklahoma passed a tribal law recognising the rights of nature as part of a movement against fracking (International Cry 2017; International Center for the Rights of Nature 2019). In May 2019, the Yurok Tribal Council in California passed the Resolution Establishing the Rights of the Klamath River, emphasising the strong connection between the Yurok Tribe and the Weron or Klamath River (Yurok Tribal Council 2019). The resolution protects the river’s rights ‘to exist, flourish, and naturally evolve; to have a clean and healthy environment free from pollutants; and to be free from contamination by genetically engineered organisms’ (Yurok Tribal Council 2019, p. 2). The resolution includes a warning to ‘[t]he United States of America, the State of California and all other entities which threaten and endanger the freshwaters, ecosystem and species of the Klamath River, that it has become necessary to provide a legal basis to protect the Klamath River, its ecosystem and species for the continuation of the Yurok people and the Tribe for future generations.’ (Yurok Tribal Council 2019, p. 2)

### Canada

Canada has also produced relational water laws, alongside campaigns by First Nations for increased control over rivers. Despite Canada having comparatively strong judicial recognition of the common law doctrine of Aboriginal title (*Calder v Attorney-General of British Colombia* (1973) 34 DLR (3rd) 145 1973; *Delgamuukw v British Colombia* 1997; *Tsilhqot’in Nation v British Colombia* 2014), Indigenous water rights are sourced in a complicated patchwork of treaties, constitutional law,^23^ fiduciary duty (*Guerin v The Queen* 1984), and native title, and are often ignored by water planners and regulators (Tsatsaros et al. 2018; Wilson 2019). First Nations continue to endure major social, economic, environmental and health injustices with respect to water access (Latchmore et al. 2018), while the water governance system perpetuates the suppression of Indigenous ways of knowing and relating to water, including those based on kinship (Yates et al. 2017; Bakker et al. 2018; Wilson and Inkster 2018).

The Canadian Federal Government has at times settled resource disputes at the regional scale via agreement-making with Indigenous peoples (James Bay and Northern Quebec Agreement of 1975; the Inuvialuit Final Agreement of 1984; Nunavut Agreement of 1999), which also involve provincial and local governments, and sometimes non-Indigenous entities (like hydro generators) (Tsatsaros et al. 2018, p. 11). These include ‘co-management’ arrangements similar to those in Aotearoa involving governments, First Nations and other water users in co-management boards, which may make decisions around water allocation and management (Burchi 2005, pp. 32–36). An example is the Cowichan Watershed Board established as part of an agreement between the Cowichan Tribes and the regional government (Curran 2019, p. 9). The Board undertakes a collaborative approach to water governance with governmental and non-governmental stakeholders and applies Indigenous custom-based principles for integrated catchment management. Yet, as is seen in Aotearoa, co-management models don’t necessarily meet the standards of genuine partnership and power-sharing in their design and implementation.

First Nations in Canada have also sought legal personhood arrangements as part of collaborative river governance approaches. In 2021, the Innu Council of Ekuanitshit and the regional municipality of Minganie passed joint resolutions declaring that the Muteshekau-shipu (Magpie River) was a legal person, with support of the International Observatory for The Rights of Nature (Noël L. Magpie River Resolution 2021). This followed Indigenous activism to protect the river from hydro development. According to media reports, the declarations grant to the river: the right to flow; the right to respect for its cycles; the right for its natural evolution to be protected and preserved; the right to maintain its natural biodiversity; the right to fulfil its essential functions within its ecosystem; the right to maintain its integrity; the right to be safe from pollution; the right to regenerate and be restored; and the right to sue (Berge 2016). The river’s status is said to be ‘rooted in the legal underpinnings of international and national law relating to First Nations’, enabling a ‘paradigm shift’ in the way rivers are managed (Pier-Olivier and Wilson 2021). However, the legal status and force of the declarations is unclear (Raymer 2021), the declarations themselves apparently not publicly available. Reports state that the declarations require river guardians to be appointed to oversee the protection of the rights recognised, and that the International Observatory for The Rights of Nature is currently working with other river communities seeking legal rights for rivers in Canada (Observatoire International des droits de la Nature [date unknown]).

### Chile

Chile is an extreme case of a neoliberal, technocratic approach to water law and policy, where, until recently, the market was assumed to be the best distributor of water access entitlements and public water governance was widely seen as unnecessary political interference with private property rights (Bauer 2004; Budds 2004; del Campo García and Sánchez Reinón 2021). Unlike the other countries discussed here, Chile has had legislation in place to recognise ancestral water rights, and fund the reallocation to Indigenous communities of rights lost to third parties, since the early 1990s (Indigenous Law 1993 art. 64).^24^ However, this was done to clarify and regularise water holdings, without any real attempt to understand Indigenous water ontologies (Macpherson 2017).

Indigenous water relationships in Chile have also been described in relational terms (Kelly 2021), with Mapuche believing that water is connected to ancestors, metaphysical entities and the broader environment (Barrera-Hernández 2005). Babidge (2016, p. 85) explains that Indigenous water interests in Chile are characterised by ‘complex waterscapes, where neither “rights” nor “values” capture the totality of Indigenous interests and processes’, including social, cultural, spiritual, economic and environmental dimensions. Yet, other than as water rights owners within river user associations (Water Code 1981 arts. 187–282), Indigenous peoples and laws have been completely absent from Chilean water governance. Meanwhile, water-related conflicts have disproportionately affected Indigenous peoples (Marin 2016, p. 91), whose relationships with and economic dependence on water are increasingly encroached upon (Camacho 2012).

That being said, Chilean water policy has come under increasing scrutiny in the past couple of years, as a citizen assembly will soon embark on a second attempt to draft a new Constitution for Chile (Ansaldi and Pardo-Vergara 2020; Hilbink 2021; Prieto and Verdugo 2021); the first draft of which was described as both a ‘green’ and ‘Indigenist’ constitution (Berasaluce et al. 2021), but failed to obtain the approval of a majority during a September 2022 plebiscite. The constitutional process builds on many years of agitation for the recognition of water as a basic human right rather than a marketable commodity. In 2022, Chile’s Water Code was amended to recognise ecosystem interests and human rights in water as priority interests (Proyecto de Ley Que Reforma El Código de Aguas). The amendment also recognised Indigenous rights to water and attempted to reverse the separation of land and water titles in the Water Code;^25^ considered inconsistent with Indigenous ways of understanding the place-based connectedness of people, land and water. However, concern about the Constitutional protection of water, and related Indigenous rights, remained (Hilbink 2021).

The first attempt at drafting a new Constitution for Chile was led by a citizen assembly, with an equal number of men and women, and 17 seats out of 155 were reserved and filled by representatives of 10 Indigenous nations. Significantly, one of these Indigenous members, Mapuche scholar Dr Elisa Loncon, was elected intial President of the Convention. The text produced by the assembly, and ultimately rejected by the Chilean nation, had many relational aspects, including a number of environmental, human, and Indigenous rights protections as well as rights of nature (Constitutional Convention of Chile 2022). The first article in the draft declared that Chile is ‘plurinational, intercultural and ecological’ Republic of solidarity, recognising as ‘intrinsic and irrevocable values ⁣⁣the dignity, liberty, and substantive equality of human beings and their indissoluble relationship with nature’. The draft adopted the principle of ‘buen vivir’ (or living well) from the Ecuadorian Constitution, based on ‘a relationship of harmonic equilibrium between people, nature and society’ (cl 9A).

The draft recognised water as a human right for current and future generations (cl 202), which it redefined as ‘common natural property’ (cl 205), which cannot be appropriated, but must be ‘preserved, conserved and restored’ by the state (cl 206). It provided that, ‘people and communities are interdependent with nature and form, with it, an inseparable connection’, and, ‘[n]ature has the right to have its existence respected and protected, to regeneration, maintenance and restoration of its dynamic functions and equilibriums, including natural cycles, ecosystems and biodiversity’ (cl.204). The State, in turn, would be required via its institutions to guarantee and promote the rights of nature.

The failed draft also included a number of provisions directed at Indigenous peoples, including a wide-ranging constitutional recognition of Indigenous rights and authority that could have had major structural implications for Chile’s historically conservative legal frameworks (proposed art. 5). This provision sought to recognise Indigenous ‘nationhood’ and self-determination, full exercise of individual and collective rights, rights to autonomy and self-government, and rights to lands and territories, institutions and jurisdictions. The provision required the state to:

… guarantee the effective participation of indigenous peoples in the exercise and distribution of power, incorporating their representation in the structure of the State, its organs and institutions, as well as its political representation in popular electoral bodies at the local, regional and national levels. Furthermore, the State will guarantee intercultural dialogue in the exercise of public functions, creating institutions and promoting public policies that support the recognition and understanding of ethnic and cultural diversity of the indigenous peoples and nations that pre-exist the State.

Debate and controversy in Chile around the Chilean constitutional reform project intensified towards the September plebiscite, including ‘push back’ around the rights and roles recognised for Indigenous peoples (Oyarzún-Serrano 2020; del Campo García and Sánchez Reinón 2021). The Chilean approach is a reminder about the power of politics and the politics of power (Bauer 2004; Budds 2004) in determining water law frameworks, including attempts to secure more relational water laws with constitutional implications, which require delicate manoeuvring.

## Discussion

What does the survey of comparative water laws across Australasia and Latin America tell us about the potential for Western water law to become more relational? As was discussed at the outset of this article, relationality has substantive and procedural implications for water law. It entails recognising ‘relational closeness’ (Arstein-Kerslake et al. 2021), belonging (Clark and Page 2022), interconnectedness and holism in relationships between people and place that determine the nature and scope of water use and care, and the necessary partnership and power-sharing between governments and water peoples to support the performance of relational governance. A relational approach to water law, therefore, is based on co-constitutive relationships and relatedness between people and place, but also between peoples.

The seven settler-colonial jurisdictions highlighted in this article are all sites of ongoing conversation and agitation around social and constitutional transformation (in one way or another), to which Indigenous and environmental justice are key. The highlights discussed here include attempts by Western legal frameworks to relate with Indigenous systems of law and governance for water, although this is not always done in a way that meaningfully respects, reflects and re-enacts relationships between water peoples and water places. As with most things, there is a spectrum of pluralism and partnership evident across global initiatives, with some legal mechanisms reflective and supportive of Indigenous ontologies and political strategies. Others are still underpinned by troubling Western dominance and assumptions.

Table 1 attempts to evaluate the comparative laws highlighted, in terms of themes discussed in this article, simplistically ranking them as ‘high’, ‘medium’ or ‘low’ in the extent to which they support or reflect elements indicative of relational water laws (see intro and part 2). These are: the extent to which the law reflects the relatedness of people and place; whether the law depends on constitutional rights protections; the extent to which the law reflects or defers to Indigenous law; the degree to which the law provides for representation arrangements; the effectiveness of institutions and collaborations to implement the law; and the extent to which the law enables power-sharing or transfers. The table is not intended to be complete or authoritative, but permits some tentative observations around the prospects for a more relational Western water law. These include the potential for constitutional law to buoy the approach, although without place-based specificity and supporting institutions, resources and distributions of power, constitutional approaches may have little impact.

## Conclusion

Coming back to Aotearoa, although subtle and incremental inroads are being made into Crown legal frameworks (Coates 2018, p. 160), water relationality is unfinished constitutional business (Ruru 2018a, p. 121). Coates (2018) signals that internal reflection and discussions are needed within Māori communities about what constitutional transformation may look like, including pragmatically-driven yet conscious reframing of its foundations. I have argued that a relational approach to water law would require partnership between the Crown and Māori, at the very least. Partnership (required by Te Tiriti) has constitutional implications because it goes to the very framing and design of legal frameworks which cannot be mere top-down directives by states that ‘involve’ or ‘engage’ peoples. It requires a wiliness to recognise legal pluralism and enable power-sharing. A Tiriti partnership approach to water law, if it is to meet the yardstick of relationality, requires much more than discrete ‘co-management’ arrangements for particular water resources, which has been applied here and elsewhere in limited legal frameworks mediated by settler governments (eg Canada, Australia). As Vertongen (2022, p. 4) reminds us, ‘constitutional beginnings are about how different governance concepts are to be balanced, intertwined, reconciled here in Aotearoa. Talking about co-governance is at the core of our constitutional whakapapa’.

It is also important to remember that not everyone is comfortable with discussing water rights or governance alongside the principle of partnership. Mikaere (2007) warns that, ‘[r]eliance on the principles of the Treaty in order to describe the relationship between Māori and the Crown leads us down the murky path of ‘partnership’ whereby tino rangatiratanga is made subject to kāwanatanga through such euphemistic contrivances as “reasonable cooperation”, “good faith” and “reciprocity”.’ Yet, Taylor (2022) asks us to ‘be mature and brave enough’ to transition to a relational way of governing water that honours and is informed by both cultures. Law can help or hinder this process (depending on who is weaponising it). But there is now a global movement towards a water governance that acknowledges and supports the relatedness within and between waters and water peoples – a governance that is not just consultation or participation, but real power-sharing. If Western water law is to become more relational, this will require a commitment by states to recognise the political authority of Indigenous peoples, and the courage to continue with constitutional conversations about water futures.

## Notes

1 An example is the claim being brought by Ngāi Tahu for rangatiratanga (political authority) over all waters in their territory (Ngāi Tahu 2020).

2 I adopt a broad and holistic understanding of ‘water law’ in this article, not just to contemplate the system of laws bearing on the allocation and use of water resources (as is traditional in natural resources law doctrine), but also other laws (such as Indigenous, planning, environment and conservation laws) that impact on the management and governance of aspects of water systems.

3 The use of ‘engage’ is interesting and appears to go further than mere consultation or participation imperatives typical of previous approaches.

4 I acknowledge, as a comparative legal scholar of Pākēhā (European settler) heritage, my work views the laws of multiple jurisdictions usually through an outsider’s lens.

5 The three spheres model was one of six possible models proposed.

6 For examples of other river settlements see Waikato-Tainui Raupatu Claims (Waikato River) Settlement Act 2010 (NZ); Ngāti Rangi Claims Settlement Bill 2018 (NZ) s. 107 (2).

7 This is reflected in the Coal Mines Amendment Act 1903 s. 14; Resource Management Act 1991 s. 354; Water and Soil Conservation Act 1967 s. 21; Water Power Act 1903 ss. 2, 5.

8 The Tribunal framed the interests of the Whanganui iwi in the river in terms of ‘ownership’ of a ‘single and indivisible entity comprised of water, banks, and bed’, and recommended the return of ownership of the river to them.

9 See Morris and Ruru (2010).

10 One representative nominated by the Crown and the other by the Whanganui iwi, with consensus decisionmaking.

11 Te Awa Tupua (Whanganui River Claims Settlement) Act 2017 (NZ) ss. 41, 46. The water in the river remains vested in the Crown on behalf of the New Zealand public, only the Crown-owned parts of the bed is vested in Te Awa Tupua, and the settlement has no impact on other public or private river uses, in a provision that expressly protects the rights of hydro-electric power generators.

12 In 2020, the Victorian Government ‘handed back’ 2 gigalitres of ‘winterfill’ water on the Mitchell River in south-eastern Victoria to Gunaikurnai Land and Waters Aboriginal Corporation. This water is under a ‘winterfill’ licence (meaning that water can only be extracted from the river during the high flow winter-spring period).

13 Indigenous territorial rights are recognised in *Constitución Política de Colombia* art. 330*.* See, generally, Macpherson (2019).

14 This was achieved as part of a constitutional action (or writ) called a *tutela*. See generally Vargas-Chaves et al. (2020); Coombe and Jefferson (2021).

15 The development of the rights of nature in Ecuador and Bolivia has also occurred within a context of legal and cultural pluralism, via the concept of ‘Buen Vivir’ – to live a good life*.* See, generally, Perreault (2017); Kauffman and Martin (2017).

16 The Amazon case actually ignores Indigenous territorial rights in the Amazon. See Amazon Case (2018).

17 Water regulation is generally a matter for State or municipal law, which Indigenous territorial rights, within the *ejido* reserve system, come within federal jurisdiction under the *Ley Agraria* (Agrarian law). See Ley de Aguas Nacionales [National Water Law] 1992 (Mexico) 1992 art. 3 XXVIII; Constitución Política de los Estados Unidos Mexicanos, art. 27.

18 There has also been case law connecting environmental rights to rights of nature. See, e.g., Vecinos Laguna del Carpintero (2018, pp. 6–7).

19 For example, in the State of Guerrero, the Ley de Equilibrio Ecológico y Protección al Ambiente (Law of Ecological Equilibrium and Protection of the Environment) makes no mention of the precautionary principle or the rights of nature. See, also, Ley de Equilibrio Ecológico y Protección al Ambiente del Estado de Guerrero [Law of Ecological Equilibrium and Environmental Protection for the State of Guerrero].

20 Under Article III, a plaintiff must establish; (1) that they suffered injury in fact, invading a legally protected interest which is (a) concrete and particularised, and (b) actual or imminent (not conjectural or hypothetical); (2) a causal relation between the injury and the conduct of the complaint, fairly traceable to the defendant’s action(s) (not an independent third party); and (3) that it is likely (not speculative) that the injury will be redressed by way of judicial decision.

21 For example the Tamaqua Borough Sewage Sludge Ordinance, p. s 5 “Land Application or Land Apply”; see also Pittsburgh Code; The City of Santa Mónica Sustainability Rights Ordinance, Santa Mónica Municipal Code. Most recently, voters in Orange County, Florida, overwhelmingly approved a charter amendment recognizing natural rights for the Wekiva River, Econlockhatchee River, and all waterways in the county (Orange County 2020).

22 In 2019, the White Earth Nation Band of Ojibwe in Minnesota passed an ordinance to protect wild rice (called Manoomin) as a central food for the continuation of their culture and identity (1855 Treaty Authority 2019). The ordinance recognizes the legal right for Manoomin “to exist, flourish, regenerate, and evolve, as well as inherent rights to restoration, recovery, and preservation.”

23 As protected by Constitution Act 1982 (CA) s. 35. See Nowlan (2004); Burchi (2005).

24 For an example of the application of this legislation to prevent the allocation of water to a commercial water bottler see *Aqua Mineral Chusmiza v Communidad Indigena e Chusmiza* (Supreme Court decision) 2009 [1].

25 Indigenous resistance to separation of land and water, in line with international and regional law and jurisprudence, has been emphasised since the development of the *Indigenous Law*. See Macpherson (2019, pp. 161–210).
